# Supplementary figures and images for: AQP1 Is Not Only a Water Channel: It Contributes to Cell Migration through Lin7/Beta-Catenin
Source: PLoS One. 2009 Jul 8;4(7):e6167. doi: 10.1371/journal.pone.0006167 (PMC2701997; doi:10.1371/journal.pone.0006167)

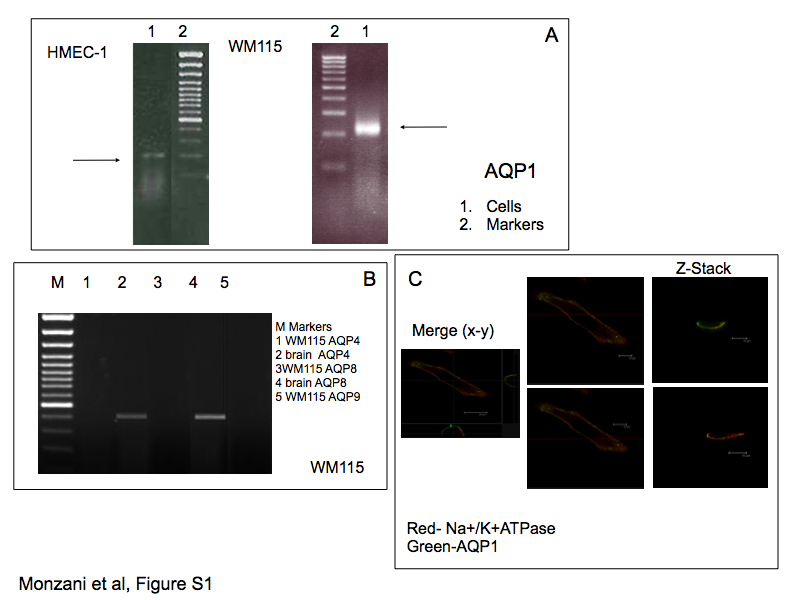

Supplement: Figure S1 — Expression of AQPs and intracellular localization. Panel A. RT-PCR of AQP1 in WM115 and HMEC-1 cells. Total RNA was purified from subconfluent cells using the RNeasy Mini Kit from Qiagen. One microgram of total RNA was reverse transcribed and amplified by Enhanced avian hs RT-PCR (Sigma) according to the manufacturer's instructions. PCR conditions and primers are reported in the Materials and Methods section. As housekeeping gene, GAPDH was used. In order to check the specificity of the amplified bands they are sequenced. Panel B. RT-PCR of AQP 8, 4 and 9 in WM115 cells for HMEC-1 and WM115 cells as described in panel A. As positive control, human brain tissue expressing AQP4 and AQP8 was used. Panel C. Co-immunofluorescence of AQP1 and Na+/K+ ATPase in WM115 cells. The cells fixed with methanol for 10 min and then permeabilized with 0.01% TRITON X-100 for 20 min, incubated with the primary antibodies (anti-AQP-1, 1∶100; mouse anti-Na+/K+ ATPase (1∶250) for 2h at room temperature. The cells were then incubated with secondary antibodies: Alexa Fluor-488 conjugated goat anti-rabbit, (1∶700, Molecular Probe), Alexa Fluor-594-conjugated goat anti-mouse, (1∶700, Molecular Probes) for 1h and examined on a Leika TCS NT confocal microscope. The localization of both proteins in plasma membrane are shown x-y and z-stack analysis. AQP1 is the main aquaporins expressed in these cells. The protein is mainly expressed at the level of plasma membrane. In order to clearly demonstrate the plasma membrane localization of AQP1, we have co-stained the cells with anti-AQP1 and anti-Na+/K+ ATPase (typical membrane marker) antibodies. As shown in panel C of figure 1, AQP1 shows a plasma membrane localization as demonstrated both by the merge and Z-stack analysis. (0.14 MB TIF) [file pone.0006167.s001.tif]

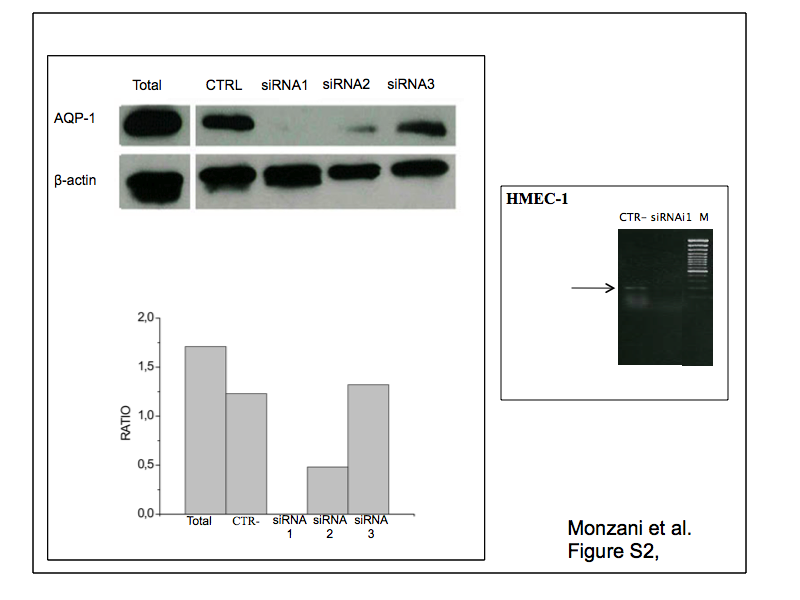

Supplement: Figure S2 — AQP1 siRNAs in WM115 and HMEC-1 cells. WM115 or HMec-1 cells were tranfected with siRNA AQP1-1, AQP1-2, AQP1-3 or negative control siRNA CTRL1 for 48 h as described in the Materials and Methods section. 50ug protein was submitted to 10% SDS-PAGE and transferred to a PVDF sheet. The latter was incubated with anti-AQP1 (1∶2000) or beta-actin (1∶5000) as housekeeping protein, for 2h. Then the sheet was incubated with secondary antibody and visualized using the ECL detection system. Densitometric analysis expressed as ratio of each line with respect to beta-actin is shown. The figure shows that siRNA1-1 is more effective to down-regulate the expression of AQP1 with respect to AQP1-2 and AQP1-3. (0.12 MB TIF) [file pone.0006167.s002.tif]
